# Independent Association of Thyroid Dysfunction and Inflammation Predicts Adverse Events in Patients with Heart Failure via Promoting Cell Death

**DOI:** 10.3390/jcdd9090290

**Published:** 2022-08-31

**Authors:** Yimin Shen, Guanzhong Chen, Sheng’an Su, Chenchen Zhao, Hong Ma, Meixiang Xiang

**Affiliations:** Department of Cardiology, The Second Affiliated Hospital, Zhejiang University School of Medicine, Hangzhou 310009, China

**Keywords:** heart failure, thyroid dysfunction, inflammation, mortality, rehospitalization

## Abstract

Thyroid dysfunction and inflammation are individually implicated in the increased risk of heart failure. Given the regulatory role of thyroid hormones on immune cells, this study aimed to investigate their joint association in heart failure. Patients with pre-existing heart failure were enrolled when hospitalized between July 2019 and September 2021. Thyroid function and inflammatory markers were measured at the enrollment. The composite of all-cause mortality or rehospitalization for heart failure were studied in the following year. Among 451 participants (mean age 66.1 years, 69.4% male), 141 incident primary endpoints were observed during a median follow-up of 289 days. TT3 and FT3 levels were negatively correlated with BNP levels (r: −0.40, *p* < 0.001; r: −0.40, *p* < 0.001, respectively) and NT-proBNP levels (r: −0.39, *p* < 0.001; r: −0.39, *p* < 0.001). Multivariate COX regression analysis revealed that FT3 (adjusted HR: 0.677, 95% CI: 0.551–0.832) and NLR (adjusted HR: 1.073, 95% CI: 1.036–1.111) were associated with adverse event, and similar results for TT3 (adjusted HR: 0.320, 95% CI: 0.181–0.565) and NLR (adjusted HR: 1.072, 95% CI: 1.035–1.110). Restricted cubic splines analysis indicated a linear relationship between T3 level and adverse events. Mechanistically, primary cardiomyocytes showed strong resistance to TNF-α induced apoptosis under optimal T3 concentrations, as evidenced by TUNEL staining, flow cytometry analysis, and LDH release assay as well as increased expression of Bcl-2. Thyroid dysfunction and inflammation are independently associated with cardiovascular risk in heart failure patients, which may concurrently contribute to the ongoing cardiomyocyte loss in the disease progression.

## 1. Introduction

Thyroid hormones (THs) play an indispensable role in development as well as organ homeostasis. They have been implicated to regulate the cardiovascular system, including cardiac rhythm, contractility, hypertrophy, and vascular resistance [1,2]. Thyroid dysfunction, even a minor alteration of circulating THs, may induce or exacerbate cardiovascular disorders towards heart failure (HF) [3]. Indeed, either hypothyroid or hyperthyroid states were identified to be related to 58% and 85% increases in cardiac death relative risk compared with euthyroid status [4]. Moreover, both overt and subclinical thyroid dysfunction can adversely affect cardiac function [3]. Most importantly, thyroid dysfunction is modifiable, which brings a potential therapeutic option for patients with heart failure. However, the significant gap concerning the exact molecular basis underlying the interaction between the thyroid and cardiovascular systems becomes a major hurdle to optimally manage patients with both thyroid and cardiac abnormalities.

In the past decades, the immune system has been regarded as an important target of THs [5]. The systematic inflammatory status is affected by TH levels. Hypothyroidism tends to suppress the activation of inflammatory response, while hyperthyroidism commonly enhances the activity of neutrophils and lymphocytes [6]. Interestingly, an ongoing inflammatory response is considered as a major regulator in the pathogenesis of heart failure [7]. Since the 1990s, elevated circulating levels of tumor necrosis factor α (TNF-α) have been noticed in HF patients [8]. Accumulating evidence has highlighted an important role of the inflammatory response, featured by accumulated circulating pro-inflammatory cytokines and immune cells in the heart, and in acute or chronic heart failure resulting from distinct etiologies [9,10,11,12]. Targeting inflammation could be a potential therapeutic utility, as revealed by the subanalysis of the CANTOS trial [13]. However, whether the prevalence or prognostic significance of thyroid dysfunction in HF is related to inflammation has not been investigated.

Interestingly, patients with chronic heart failure may present with overt or subclinical hypothyroidism [14]. The increase of proinflammatory cytokines such as TNF-α have been noticed to be associated with low-T3 syndrome [15], which may result from the adverse effects of inflammation on the conversion of thyroxine (T4) to triiodothyronine (T3). However, hypothyroidism seems to undermine the pro-inflammatory role of neutrophils, macrophages, and lymphocytes, which is supposed to be beneficial to heart failure. On the contrary, previous studies showed that administration of low levels of T3 can promote cardiac function to some extent [16,17]. Little is known about the interconnection among thyroid dysfunction and inflammation in HF. To address this important gap, we aimed to explore the joint association of thyroid dysfunction and inflammation with cardiovascular risk in heart failure patients. We hypothesized that these two factors may simultaneously promote ongoing cardiomyocyte loss, leading to adverse events in heart failure.

## 2. Materials and Methods

### 2.1. Study Population

**Figure 1:**
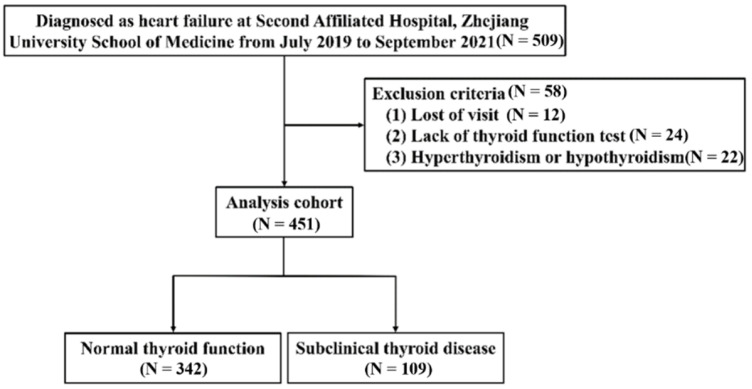
Enrollment and outcomes.

From July 2019 to September 2021, 509 patients hospitalized with HF in the Second Affiliated Hospital, Zhejiang University School of Medicine, with complete clinical data and which provided written informed consent were enrolled. HFs were defined based on the 2022 heart failure guidelines [18]. Patients without echocardiogram, lack of thyroid function test, or loss of visit were excluded. Patients with hyperthyroidism or hypothyroidism were also excluded. Generally, Probable HF hospitalizations (*n* = 509) were eligible for inclusion. Fifty-eight patients met the exclusion criteria, and 12 patients were lost to follow-up. Finally, 451 validated HF patients were included (Figure 1). The study was conducted on the grounds of the Declaration of Helsinki and approved by the Ethics Committee of the Second Affiliated Hospital, Zhejiang University School of Medicine.

### 2.2. Thyroid Hormone Sampling and Subgroups of Thyroid Status Definition

We used electrochemiluminescence Immunoassay (ECLIA) for the analysis of THs immediately after sampling. The reference ranges were thyrotropin (TSH), 0.35 to 4.94 mIU/L, free thyroxine (FT4), 9.01 to 19.05 pmol/L. Further categorical analysis assigned patients to the following groups based on FT4, free triiodothyronine (FT3), and TSH levels through our institution’s cutoffs. Euthyroidism refers to TSH of 0.35 to 4.49 mIU/L; subclinical hypothyroidism (SCH) refers to TSH of 4.94 to 19.9 mIU/L with normal FT4; subclinical hyperthyroidism refers to TSH levels under the reference range with normal FT4 levels; additionally, low-T3 syndrome refers to TSH of 0.35 to 4.94 mIU/L with decreased FT3 [19].

### 2.3. Data Collection and Clinical Outcome

For patients with multiple admission records, the latest medical record was adopted as the baseline record. Clinical status, comorbidities, medication, intervention, laboratory results, and echocardiogram results were obtained from this clinical database. Echocardiogram results were left ventricular ejection fraction (LVEF), left ventricular end-diastolic volume (LVEDV), interventricular septum thickness in diastolic phase (IVSd), left atrium (LA) dimension (defined as the largest diameter of left atrium in parasternal long axis view), left ventricular internal diameter in diastolic phase (LVIDd) and left ventricular posterior wall thickness in diastolic phase (LVPWd). Laboratory results include brain natriuretic peptide (BNP), N-terminal pro-B type natriuretic peptide (NT-proBNP), cardiac troponin T (cTnT), kinase isoenzyme-MB (CK-MB), CRP, full blood counts (like neutrophils, lymphocytes, monocytes), creatine (Cr), hemoglobin (Hb), alanine aminotransferase (ALT), glycated hemoglobin (HbA1c), serum glucose (Glu), and triglycerides (TG). The quality of medical therapy in heart failure was measured by Heart Failure Collaboratory (HFC) score. The quality of medical therapy was considered as sub-optimal, acceptable, and optimal treatment when the score was <3, 3–4, and ≥5, respectively [20]. The primary endpoint was the composite of all-cause mortality or rehospitalization for heart failure.

### 2.4. Cell Culture

Neonatal rats (1 to 3 day old) were supplied by the Zhejiang province animal center, China. A neonatal heart dissociation kit (No 130-098-373, Miltenyi Biotec, Bergisch Gladbach, Germany) was utilized for the primary neonatal rat ventricular cardiomyocytes prepared. Cells were cultured in a complete medium with 10% FBS, 1% penicillin-streptomycin, and Brdu (1:200), and then incubated in a 37 °C humidified incubator with 5% CO_2_. T3 was provided by Sigma (No T074, St. Louis, MO, USA) and cells were treated with 1, 10, 30, 60, and 120 ng/mL T3 as well as 10 ng/mL TNF-α for 24 h to induce an inflammatory response based on previous studies [21,22].

### 2.5. Cell Apoptosis Assay and Apoptosis Detection by Western Blot

Apoptotic cardiomyocytes were detected by TUNEL labeling (Beyotime, Institute of Biotechnology, Haimen, China). Nuclei were stained by DAPI, and TUNEL-positive cells were quantified by the high content-screening method. The percentage of apoptotic cells was considered as the apoptotic index, and each group was randomly chosen by 16 fields of each well. Meanwhile, AnnexinV-FITC/PI Apoptosis Detection Kit (MultiSciences Biotechnology) was used for detecting cardiomyocytes apoptosis based on flow cytometry. In the LDH release assay, cells were planted in a 96-well plate, and treated with TNF-α along with various concentrations of T3 (0 ng/mL, 10 ng/mL, 30 ng/mL, 60 ng/mL and 120 ng/mL) for 24 h. Then cells were managed by the LDH Cytotoxicity Assay kit (Beyotime, Institute of Biotechnology, Haimen, China) and determined under the absorbance at 490 nm. SDS-PAGE gels and PVDF membranes were used for the protein separation and transferring respectively (Millipore corporation, Shanghai, China). Five percent milk was prepared for the membranes blocked for 60 min before taken to the antibodies against Bcl-2 (1:500 dilution) and Bax (1:1000 dilution) at 4 °C temperature overnight. The membranes were then washed by 1 xPBST 3 times and taken with anti-Rabbit secondary antibodies for 60 min. β-actin served as a loading control.

### 2.6. Statistical Analysis

Continuous variables in both thyroid dysfunction and euthyroid groups were tested for the normal distribution. The results were presented as number (%), mean ± standard deviation (if data fitted normal distribution), or median ± quartile (if data did not fit normal distribution). Categorical variables were compared among groups using chi-squared test or Fisher’s exact test. Continuous variables were compared using unpaired Student’s t-test or Kruskal-Wallis’s test as appropriate. Univariate and multivariate COX regression analysis was constructed to investigate the association of thyroid function and inflammation values with the primary endpoint. Cubic splines were taken to evaluate the linearity between T3 and the incidence of study endpoints. Quantitative data are presented as the mean ± standard deviation of three independent experiments. *p* values <0.05 were considered statistically significant. The R package and python were used for all statistical analyses with two-tailed *p* values of 0.05.

## 3. Results

### 3.1. Study Subjects and Baseline Characteristics

Four hundred and fifty-one participants (69.40% males) with a median age of 66 years old were finally included in this study. Table 1 shows the baseline characteristics of the study population. Among the 451 patients included, 109 patients (24.17%) were classified as having thyroid dysfunction. In the thyroid dysfunction group, cardiac function parameters including NT-proBNP, BNP, and cTnT levels were nearly two-fold higher. FT4, total thyroxine (TT4), FT3, and total triiodothyronine (TT3) levels were lower in the thyroid dysfunction group, whereas TSH, as well as TPOAb levels, were higher in those patients.

### 3.2. Correlation of Thyroid Function with Cardiac Parameters and Inflammation Values

**Figure 2:**
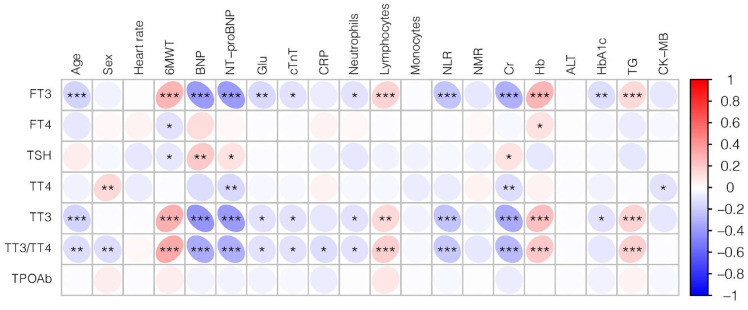
The correlation between thyroid hormones, inflammation values, and cardiac parameters in HF patients. Negative correlations are shaded blue, while positive correlations are shaded red. The strength of the correlation is indicated by dot gradient and color saturation. * *p* < 0.05, ** *p* < 0.01, *** *p* < 0.001. Abbreviations: FT3, Free triiodothyronine; FT4, Free thyroxine; TSH, Thyrotropin; TT4, Total thyroxine; TT3, Total triiodothyronine; 6MWT, The six minute walking test; BNP, Brain natriuretic peptide precursor; NT-proBNP, N-terminal (NT)-pro hormone BNP; Glu, Glucose; cTnT, Cardiac troponin T; CRP, C-reactive protein; NLR, Neutrophil-to-lymphocyte ratio; NMR, Neutrophil-to-monocyte ratio; Cr, creatinine; Hb, Hemoglobin; ALT, Alanine aminotransferase; HbA1c, Hemoglobin A1c; TG, Triglyceride; CK-MB, Creatine kinase-MB.

As baseline, characters exhibited a significant difference of HF biomarkers, such as BNP and NT-proBNP based on thyroid status; we then sought to detect the association between THs and cardiac function. A significant trend to a negative association was also present in the TT3 levels with BNP (r: −0.40, *p* < 0.001) and NT-proBNP (r: −0.39, *p* < 0.001), respectively. Next, we assessed the correlation between inflammation values and thyroid function. There were negative correlations between NLR as well as neutrophils with FT3 (r: −0.22, *p* < 0.001; r: −0.11, *p* = 0.022, respectively) and TT3 values (r: −0.23, *p* < 0.001; r: −0.12, *p* = 0.014, respectively) (Figure 2).

### 3.3. Relationship of Thyroid Function and Inflammation Values with Adverse Events

**Figure 3:**
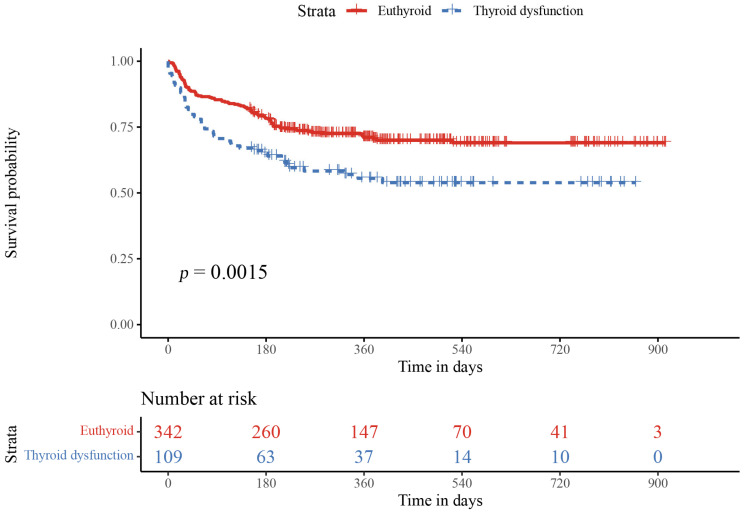
Kaplan–Meier survival curves for thyroid dysfunction compared with the euthytoidism group. Survival probability rate based on the thyroid function in patients with enrolled subjects. The red line represents the survival probability rate of the euthyroid group, while the blue line represents the thyroid dysfunction group. A significant difference was found between the two groups (*p* = 0.0015).

**Figure 4:**
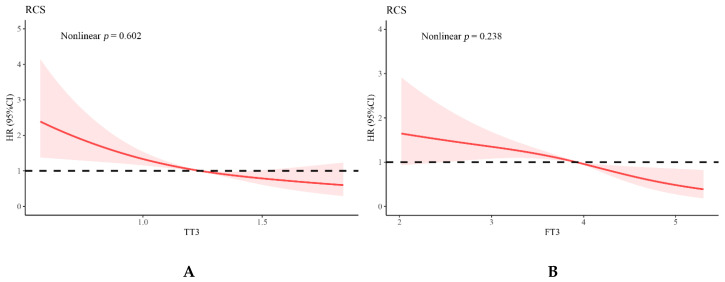
Cubic spline depicting the association of T3 with the incidence of composite of all-cause mortality or rehospitalization for heart failure (adjusted for age, sex, Amiodarone use, BMI, and LVEF). (**A**). TT3, *p* for non-linear = 0.602, *p*-overall < 0.0001; (**B**). FT3, *p* for non-linear = 0.238, *p*-overall < 0.0001. Abbreviations: TT3, Total triiodothyronine; FT3, Free triiodothyronine; BMI, Body mass index; LVEF, Left ventricular ejection fraction.

Over a median follow-up period of 289 days (interquartile range, 161–485 days), the rate of the adverse events (all-cause mortality or HF rehospitalization) was 31.3% (*n =* 141). Kaplan–Meier analysis revealed that prognosis in patients with thyroid dysfunction was worse (*p* = 0.015) (Figure 3). Univariate COX regression analysis was first adopted to recognize statistically significant factors with elevated risks of composite cardiovascular events. With adjustment for age, sex, Amiodarone use, body mass index (BMI), and LVEF, FT3 (adjusted HR: 0.677, 95% CI: 0.551–0.832, *p* < 0.001) and NLR (adjusted HR: 1.073, 95% CI: 1.036–1.111, *p* < 0.001) were independently associated with adverse events. Similarly, TT3 (adjusted HR: 0.320, 95% CI: 0.181–0.565, *p* < 0.001) and NLR (adjusted HR: 1.072, 95% CI: 1.035–1.110, *p* < 0.001) were also independently associated with adverse events after adjusting for age, sex, Amiodarone use, BMI, and LVEF (Table 2). Assessment of cubic splines also supports a linear relationship between T3 and the incidence of study endpoints (TT3, *p* for nonlinear = 0.602, *p*-overall < 0.0001; FT3, *p* for nonlinear = 0.238, *p*-overall < 0.0001) (Figure 4). However, there was no significant interaction between NLR with TT3 (*p* for interaction *=* 0.084) or with FT3 (*p* for interaction = 0.397) (Supplementary Table S1).

### 3.4. The Protecting Role of T3 in TNF-α Induced Neonatal Rat Ventricular Cardiomyocyte Apoptosis

**Figure 5:**
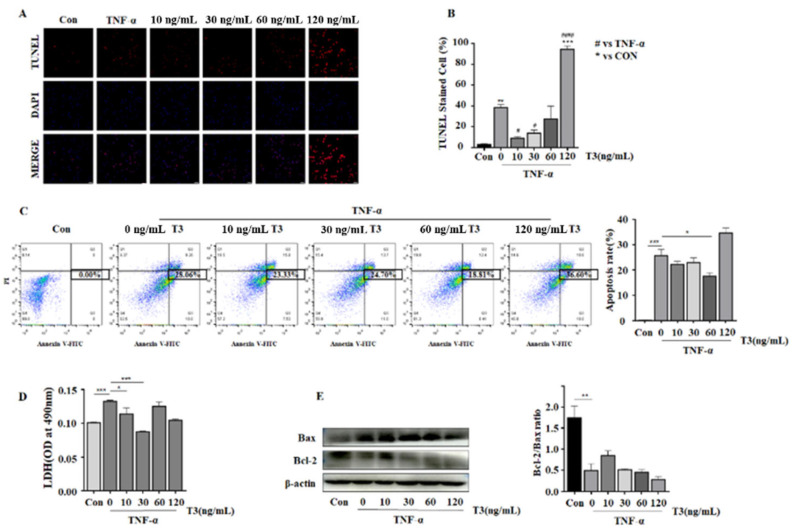
T3 protects neonatal rat cardiomyocytes against TNF-α induced cell apoptosis. (**A**). Staining of TUNEL in cardiomyocytes treated with TNF-α alone or with various concentrations of T3 (10 ng/mL, 30 ng/mL, 60 ng/mL, 120 ng/mL) for 24 h. Red spots indicate TUNEL-positive cardiomyocytes. Scale bar, 50 um. (**B**). Bar chart representing the significant changes in the percentage of TUNEL-stained cells (*n =* 3). Data are presented as the mean ± SEM. * *p* < 0.05 versus control group, ^#^ *p* < 0.05 versus TNF-α group evaluated by one-way analysis. ** *p* < 0.01, *** *p* < 0.005, ^#^ *p* < 0.05, ^###^ *p* < 0.005. (**C**). Flow cytometry analysis of apoptosis by Annexin V-FITC/PI double staining and statistical analysis of apoptosis rate. Data are presented as the mean ± SEM. * *p* < 0.05, *** *p* < 0.005. (**D**). LDH release assay with cardiomyocytes treated with TNF-α and in presence or absence of T3 (0 ng/mL, 10 ng/mL, 30 ng/mL, 60 ng/mL and 120 ng/mL) for 24 h. Data are presented as the mean ± SEM. * *p* < 0.05, *** *p* < 0.005. (**E**). Western blot assay was used for detection cell apoptotic protein Bcl-2 and Bax expression and statistical analysis for Bcl-2/Bax ratio. Data are presented as the mean ± SEM. ** *p* < 0.01.

The TUNEL method was utilized to explore the functional role of T3 on cardiomyocytes apoptosis. The cardiac apoptosis index was significantly increased compared with the control group when exposed to TNF-α. When the cardiomyocytes were further exposed to T3 with various concentrations, the apoptotic index first increased at 10 ng/mL and decreased dramatically at 30 ng/mL compared with TNF-α alone. The result indicated that 30 ng/mL of T3 alleviated cardiomyocyte apoptosis obviously (Figure 5A,B). Consistently, the flow cytometry analysis with Annexin V-FITC/PI double staining also suggested that a proper concentration of T3 supplementation may decrease cardiomyocyte apoptosis compared with TNF-α alone, whereas too low or too high T3 may not exert beneficial effects on cardiomyocytes under TNF-α stimulation (Figure 5C). LDH release assay was used to detect cell damage among each group. As shown in the Figure 5D, cells treated with TNF-α and 30ng/mL T3 significantly reduced LDH release, which consists with the TUNEL results. The expression of Bcl-2 and Bax were then determined by TNF-α or with various concentrations of T3 (10, 30, 60, and 120 ng/mL) for 24 h. We found an increased Bcl-2 protein expression when pretreated with T3, especially at 10 ng/mL, compared with the TNF-α only. These results indicated that low concentrations of T3 could prevent TNF-α-induced cardiomyocyte apoptosis (Figure 5E).

## 4. Discussion

It has been previously studied that hospitalized HF patients can suffer transient thyroid dysfunctions such as subclinical thyroid dysfunction or low T3 syndrome [23]. SCH is considered to have an increased TSH level above the upper reference limit with a normal level of serum T4 and T3 [24]. Meanwhile, low-T3 syndrome is characterized by decreased serum T3 levels with serum TSH levels in the normal range, and changes of T3 concentration can reflect the severity of the illness [25]. The presence of low-T3 syndrome or SCH has been reported following pathologic response to acute illness such as pneumonia or myocardial infarction [26,27]. Additionally, a higher risk of developing thyroid dysfunction was detected in patients with more advanced stages of HF [28]. In the present, our study investigated thyroid function changes in the pre-existing HF subjects and suggested that lower levels of TSH as well as TT3 were found associated with more severe symptoms of HF at baseline. Further analysis indicated that inflammation plays a vital role in the HF-mediated thyroid dysfunction. A possible explanation for the inverse relationship between TSH level and inflammatory response can be attributed to the higher degree of cardiac decompensation mediated endogenous stress in HF patients. Increased endogenous stress accounts for higher levels of cortisol, which further reduces TSH concentrations [29]. As a positive loop, decreased TSH concentrations resulted in higher adrenergic activity and heart rates which could, in turn, aggravate cardiac function [30]. On the other hand, HF status could lead to the transient increase in pro-inflammatory factors, like IL-1 and TFN-α, accounting for temporary changes in peripheral as well as central THs by the role of TSH on the thyroid, and, subsequently, resulting in decreased T_3_ and T_4_ levels in circulation [31].

Besides, in the setting of HF status, neutrophils accumulation acts as the initial inflammatory response followed by the infiltration of mononuclear phagocytes, including monocytes, macrophages, and dendritic cells. Neutrophils are responsible for the maintenance of inappropriate immune responses and can destroy tissue through the enzyme myeloperoxidase (MPO) and free radicals in the process of inflammation and immunity [32]. After one week, increased lymphocytes involved in the responses of the immune system, including T cells, B cells and macrophages present with anti-inflammatory effects (express IL-10) appear around this time point for inflammation resolution and LV remodeling [33]. The link between immune cells and THs metabolism was first noticed in the 1970s when radioactively labeled THs was found to appeal to the sites of bacterial infection [34]. Recently, based on the strong immunostaining of the TH-inactivating deiodinase DIO3 detected in infiltrating leukocytes in bacterial infection mice models, neutrophils are confirmed to metabolize THs [35]. Furthermore, in the cerebral spinal fluid of bacterial meningitis patients, there is a marked change of THs level characterized by increased T4 and rT3 concentrations, which is consistent with elevated DIO3 activity detected in infiltrating neutrophils [36]. Besides, lymphocytes like T cells and B cells could induce autoimmune thyroiditis and decreased plasma THs level by producing antibodies against thyroid antigens [37]. According to the above study, the correlation between THs with immune cells can be explained by either the deiodination role or the direct immunoinflammatory injury [38].

Meanwhile, the subtle changes in thyroid function may be more remarkable in pre-existing HF patients [19]. A previous study by Tomohiro et al. based on 274 subjects demonstrated that SCH or an increased TSH level could independently predict hospitalization for worsening HF [15]. Besides, in a prospective cohort study with 1365 pre-existing HF patients, patients with isolated low T3 levels or SCH with TSH ≥ 7 mIU/L indicate poorer prognosis as well [19]. Both neutrophils and lymphocytes have been determined to be strongly and independently associated with advanced HF patients’ hospitalization, survival, and survival free from heart transplant [39]. However, in patients with decompensated AHF, Uthamalingam et al. showed that NLR rather than absolute neutrophil or lymphocyte counts independently was related to the in-hospital mortality and post-discharge clinical outcomes independent of the LV function [40]. Additionally, as complete blood count involves automatic leukocyte subsets distribution analysis and is widely utilized, NLR values can be a great tool in the disease survival or prognosis assessment, probably more efficient than CRP [32]. NLR has already been used to determine the disease severity in thyroiditis, CVD, malignancies, and other inflammatory-related diseases [32,41,42,43]. Our univariate COX analysis suggested that NLR (HR: 1.090, 95% CI: 1.060–1.130, *p* < 0.001) was significantly associated with an elevated risk of composite cardiovascular events in included subjects, and the multivariate analysis showed the same results. However, the follow-up period of the present study is still not long enough, which may cause difficulties in predicting the influence of thyroid hormones and inflammatory markers on the prognosis of HF patients in long run. Further studies with longer follow-up durations are needed.

Inflammatory cytokine TNF-α plays a vital role in cell apoptosis stimulation and extracellular matrix degradation. In our study, TUNEL-positive cells and the release of LDH were increased in cardiomyocytes stimuli with TNF-α, which was decreased by the low concentrations of T3 (30 ng/mL). Apoptosis is characterized by cell shrinkage, DNA fragmentation, chromatin condensation, and apoptotic bodies [44]. Ferroptosis, an iron-dependent regulated cell death [45], can cause ROS and lipid peroxidation production [46]. Unlike typical apoptosis and autophagy, ferroptosis presented with reduced mitochondrial volume in morphology, increased density of the mitochondrial membrane, and intracellular iron content [47]. Recently, endoplasmic reticulum stress (ERS) has aroused much attention in the ferroptosis progression, which is a kind of warner under the endoplasmic reticulum dysfunction circumstance by activating the transcription factor 4-C/EBP homologous protein (ATF4-CHOP) pathway [48]. During the process of ferroptosis in mitochondria, iron ions and NADPH oxidase interaction can cause ROS production [49], which is the major stimulator for ERS [50]. Liang et al. suggested that in insulin-resistant *β*-cells, the expression of ERS apoptotic-related proteins, such as CHOP as well as caspase-12, can be upregulated by high levels of T3, accounting for cell apoptosis through ERS [51]. According to this, a lower concentration of T3 in protecting cell apoptosis maybe attributed to inactive ERS.

Additionally, we demonstrated that TNF-α could down-regulate Bcl-2 expression in neonatal rat ventricular cardiomyocytes. Cell fate is always influenced by the apoptosis-related signals, for example, Bcl-2 family members [52]. In response to deleterious events, the Bax can be activated and exhibit an amino acid homology and form heterodimers with Bcl-2 in the mitochondrial outer membrane to present with a pro-apoptotic function [53].

## 5. Conclusions

Our study suggested that HF status-mediated inflammatory responses could lead to thyroid dysfunction, which further aggravates HF progression. Additionally, low T3 concentrations could alleviate cardiomyocyte apoptosis. Besides, NLR can be a strong and independent poor predictive marker in thyroid dysfunction patients with pre-existing HF.

## Figures and Tables

**Table 1:** Baseline characteristics of the included patients according to thyroid function status.

	Euthyroid (*N* = 342)	Thyroid Abnormal (*N* = 109)	*p*-Value
Demographic characteristics			
Age (years)	64.79 [54.11; 72.77]	68.99 [58.44; 78.10]	0.004
Male, n (%)	236 (69.01%)	77 (70.64%)	0.839
Smoke, n (%)	62 (18.13%)	18 (16.51%)	0.810
Drink, n (%)	60 (17.54%)	14 (12.84%)	0.315
Physical examination and Coexisting conditions			
BMI (kg/m^2^)	23.88 [21.78; 26.57]	22.04 [19.43; 24.38]	<0.001
Heart rate (bpm)	78.00 [69.00; 87.00]	75.00 [65.00; 87.00]	0.113
NYHA class			0.002
I	42 (12.28%)	6 (5.50%)	
II	223 (65.20%)	60 (55.05%	
III	70 (20.47%)	36 (33.03%)	
IV	7 (2.05%)	7 (6.42%)	
Hypertension, n (%)	160 (46.78%)	49 (44.95%)	0.823
Diabetes, n (%)	93 (27.19%)	33 (30.28%)	0.616
Coronary artery disease, n (%)	113 (33.04%)	36 (33.03%)	1.000
AF, n (%)	115 (33.63%)	37 (33.94%)	1.000
Stroke, n (%)	22 (6.43%)	14 (12.84%)	0.051
6MWT (m)	408.00 [285.00; 485.00]	340.00 [100.00; 441.25]	<0.001
Laboratory examination			
FT3 (pmol/L)	4.12 [3.67; 4.45]	3.07 [2.48; 3.65]	<0.001
FT4 (pmol/L)	13.68 [12.57; 14.97]	13.12 [11.98; 14.96]	0.039
TSH (mIU/L)	1.61 [1.11; 2.34]	2.33 [0.74; 5.49]	0.003
TT4 (nmol/L)	103.46 (18.03)	93.49 (24.11)	<0.001
TT3 (nmol/L)	1.31 (0.23)	0.96 (0.36)	<0.001
TT3/TT4 (×10^−2^)	1.30 (0.27)	1.05 (0.35)	<0.001
TPOAb (IU/mL)	0.55 [0.50; 1.09]	0.75 [0.50; 1.55]	0.019
BNP (pg/mL)	494.00 [189.60; 1177.00]	908.60 [459.55; 3386.80]	0.001
NT-proBNP (pg/mL)	1027.00 [491.50; 2271.75]	2474.00 [932.00; 6501.50]	<0.001
GLU (mmol/L)	5.56 [4.93; 6.70]	5.64 [4.93; 6.81]	0.674
cTnT (ug/L)	0.02 [0.02; 0.04]	0.04 [0.02; 0.12]	<0.001
CRP (mg/L)	5.35 [5.00; 11.60]	7.55 [5.00; 14.78]	0.080
Neutrophils (×10^9^/L)	4.24 [3.21; 5.50]	4.15 [3.08; 5.58]	0.655
Lymphocytes (×10^9^/L)	1.41 [1.09; 1.75]	1.17 [0.90; 1.60]	0.001
Monocytes (×10^9^/L)	0.44 [0.34; 0.57]	0.43 [0.34; 0.57]	0.930
NLR	3.08 [2.13; 4.29]	3.41 [2.29; 5.09]	0.110
NMR	9.69 [7.34; 11.86]	9.35 [7.39; 11.88]	0.833
Creatinine (umol/L)	80.00 [67.00; 97.00]	90.00 [74.00; 122.00]	<0.001
Hb (g/L)	137.00 [121.00; 150.00]	125.00 [106.00; 142.50]	<0.001
ALT (U/L)	26.00 [21.00; 33.75]	31.00 [22.00; 43.00]	0.016
HbA1c (%)	6.10 [5.70; 6.80]	6.10 [5.80; 6.68]	0.851
TG (mmol/L)	1.11 [0.83; 1.48]	0.97 [0.80; 1.29]	0.045
CK-MB (U/l)	12.00 [8.00; 15.00]	11.00 [9.00; 15.75]	0.615
Medications			
ACEI/ABR/ARNI, n (%)	289 (84.50%)	78 (71.56%)	0.004
Beta-blocker, n (%)	270 (78.95%)	72 (66.06%)	0.009
Digoxin, n (%)	61 (17.84%)	24 (22.02%)	0.406
Amiodarone, n (%)	68 (19.88%)	13 (11.93%)	0.082
CCB, n (%)	27 (7.89%)	8 (7.34%)	1.000
Statin, n (%)	199 (58.19%)	58 (53.21%)	0.422
HFC score	4.00 [2.00; 4.00]	3.00 [2.00; 4.00]	0.180
Echocardiogram parameters			
LVEDV (mL)	158.60 [120.50; 208.00]	147.00 [105.40; 189.00]	0.067
LVEF (%)	33.55 [26.00; 42.38]	34.00 [27.10; 46.20]	0.190

Data presented as median [quartile] or n (%).

**Table 2:** Univariable and multivariable COX regression analyses for independent risk factors of adverse hospital events in included subjects.

Variable	Univariable		Multivariable			
	HR (95%CI)	*p*-Value	Model 1		Model 2	
			HR (95%CI)	*p*-Value	HR (95%CI)	*p*-Value
Sex	1.090 (0.767–1.540)	0.636	1.084 (0.756–1.552)	0.662	1.116 (0.778–1.601)	0.551
Age	1.020 (1.000–1.030)	0.008	1.015 (1.001–1.029)	0.033	1.015 (1.001–1.029)	0.037
BMI	0.959 (0.920–1.000)	0.048	0.992 (0.950–1.349)	0.719	0.993 (0.951–1.037)	0.758
Smoke	0.771 (0.490–1.210)	0.262				
Drink	0.737 (0.455–1.190)	0.215				
Amiodarone	0.845 (0.541–1.320)	0.459	0.853 (0.539–1.349)	0.496	0.840 (0.531–1.328)	0.456
FT3	0.597 (0.493–0.724)	<0.001	0.677 (0.551–0.832)	<0.001		
FT4	1.020 (0.937–1.120)	0.605				
TSH	1.130 (1.060–1.190)	<0.001				
TT4	0.995 (0.987–1.000)	0.220				
TT3	0.224 (0.131–0.382)	<0.001			0.320 (0.181–0.565)	<0.001
TPOAb	0.995 (0.989–1.000)	0.108				
Neutrophils	1.110 (1.030–1.200)	0.010				
Lymphocytes	0.662 (0.481–0.911)	0.011				
Monocytes	1.450 (0.697–3.020)	0.319				
NLR	1.090 (1.060–1.130)	<0.001	1.073 (1.036–1.111)	<0.001	1.072 (1.035–1.110)	<0.001
NMR	1.030 (0.997–1.070)	0.069				
HFC score	1.075 (0.963–1.199)	0.197				
LVEDV	1.000 (0.997–1.000)	0.871				
LVEF	0.990 (0.978–1.003)	0.150	0.987 (0.973–1.001)	0.075	0.988 (0.973–1.002)	0.091

Multivariate COX proportional hazards models for composite cardiovascular events (adjusting for age, sex, Amiodarone use, HFC score, BMI, and LVEF). Model 1 was constructed based on inclusion of FT3 as a continuous variable; model 2 was constructed based on the inclusion of TT3 as a categorical variable.

## Data Availability

The raw data supporting the conclusions of this article will be made available by the authors, without undue reservation.

## Supplementary Materials

The following supporting information can be downloaded at: https://www.mdpi.com/article/10.3390/jcdd9090290/s1, Table S1: Interaction analysis of NLR, TT3 and FT3.

## Abbreviations

ALT	Alanine transaminase
AF	Atrial fibrillation
ATF4	Activating transcription factor 4
BNP	Brain natriuretic peptide precursor
BMI	Body mass index; CRP, C-reactive protein
CK-MB	kinase isoenzyme-MB
cTnT	Cardiac troponin T
Cr	Creatinine
CAD	Coronary artery disease
CHOP	C/EBP homologous protein
ECLIA	Electrochemiluminescence Immunoassay
ERS	Endoplasmic reticulum stress
FT4	Free thyroxine
FT3	Free triiodothyronine
GLU	Glucose
HF	Heart failure
HFC	Heart Failure Collaboratory
Hb	Hemoglobin
HbA1c	Glycated hemoglobin
IVSd	Interventricular septal dimension
LVEF	Left ventricular ejection fraction
LA	Left atrium
LVEDV	Left ventricular end-diastolic volume
LVIDd	Left ventricular internal diameter in diastolic phase
LVPWd	Left ventricular posterior wall thickness in diastolic phase
MPO	Myeloperoxidase; NLR, Neutrophil-to-lymphocyte ratio
NT-proBNP	N-terminal pro-B type natriuretic peptide
RCS	Restricted cubic splines
SCH	Subclinical hypothyroidism
TH	Thyroid hormone
TNF-α	Tumor necrosis factor α
T4	Thyroxine
T3	Triiodothyronine
TG	Triglyceride
TT4	Total thyroxine
TT3	Total triiodothyronine
TSH	Thyrotropin
NYHA	New York Heart Association
6MWT	The six minute walking test
NMR	Neutrophil to monocyte ratio
ACEI	Angiotensin-converting enzyme inhibitor
ARB	Angiotensin receptor blocker
ARNI	angiotensin receptor neprilysin inhibitor
HR	Hazard ratio
CI	Confidence interval

## References

[1] Gerdes A.M., Ojamaa K. (2016). Thyroid Hormone and Cardioprotection. Compr. Physiol..

[2] Kalra S., Aggarwal S., Khandelwal D. (2021). Thyroid Dysfunction and Dysmetabolic Syndrome: The Need for Enhanced Thyrovigilance Strategies. Int. J. Endocrinol..

[3] Cappola A.R., Desai A.S., Medici M., Cooper L.S., Egan D., Sopko G., Fishman G.I., Goldman S., Cooper D.S., Mora S. (2019). Thyroid and Cardiovascular Disease: Research Agenda for Enhancing Knowledge, Prevention, and Treatment. Thyroid.

[4] Mitchell J.E., Hellkamp A.S., Mark D.B., Anderson J., Johnson G.W., Poole J.E., Lee K.L., Bardy G.H. (2013). Thyroid function in heart failure and impact on mortality. JACC Heart Fail..

[5] Montesinos M.D.M., Pellizas C.G. (2019). Thyroid Hormone Action on Innate Immunity. Front. Endocrinol..

[6] De Vito P., Incerpi S., Pedersen J.Z., Luly P., Davis F.B., Davis P.J. (2011). Thyroid hormones as modulators of immune activities at the cellular level. Thyroid.

[7] Dick S.A., Epelman S. (2016). Chronic Heart Failure and Inflammation: What Do We Really Know?. Circ. Res..

[8] Levine B., Kalman J., Mayer L., Fillit H.M., Packer M. (1990). Elevated circulating levels of tumor necrosis factor in severe chronic heart failure. N. Engl. J. Med..

[9] Wang L., Liang D., Xu X., Jin J., Li S., Tian G., Gao Z., Liu C., He Y. (2017). The prognostic value of neutrophil to lymphocyte and platelet to lymphocyte ratios for patients with lung cancer. Oncol. Lett..

[10] Zhang Y., Bauersachs J., Langer H.F. (2017). Immune mechanisms in heart failure. Eur. J. Heart Fail..

[11] Keliher E.J., Ye Y.X., Wojtkiewicz G.R., Aguirre A.D., Tricot B., Senders M.L., Groenen H., Fay F., Perez-Medina C., Calcagno C. (2017). Polyglucose nanoparticles with renal elimination and macrophage avidity facilitate PET imaging in ischaemic heart disease. Nat. Commun..

[12] Watts J.A., Zagorski J., Gellar M.A., Stevinson B.G., Kline J.A. (2006). Cardiac inflammation contributes to right ventricular dysfunction following experimental pulmonary embolism in rats. J. Mol. Cell. Cardiol..

[13] Ridker P.M., Thuren T., Zalewski A., Libby P. (2011). Interleukin-1beta inhibition and the prevention of recurrent cardiovascular events: Rationale and design of the Canakinumab Anti-inflammatory Thrombosis Outcomes Study (CANTOS). Am. Heart J..

[14] Chaker L., Razvi S., Bensenor I.M., Azizi F., Pearce E.N., Peeters R.P. (2022). Hypothyroidism. Nat. Rev. Dis. Primers.

[15] Hayashi T., Hasegawa T., Kanzaki H., Funada A., Amaki M., Takahama H., Ohara T., Sugano Y., Yasuda S., Ogawa H. (2016). Subclinical hypothyroidism is an independent predictor of adverse cardiovascular outcomes in patients with acute decompensated heart failure. ESC Heart Fail..

[16] Henderson K.K., Danzi S., Paul J.T., Leya G., Klein I., Samarel A.M. (2009). Physiological replacement of T3 improves left ventricular function in an animal model of myocardial infarction-induced congestive heart failure. Circ. Heart Fail..

[17] Forini F., Kusmic C., Nicolini G., Mariani L., Zucchi R., Matteucci M., Iervasi G., Pitto L. (2014). Triiodothyronine prevents cardiac ischemia/reperfusion mitochondrial impairment and cell loss by regulating miR30a/p53 axis. Endocrinology.

[18] Writing Committee Members, ACC/AHA Joint Committee Members (2022). 2022 AHA/ACC/HFSA Guideline for the Management of Heart Failure. J. Card. Fail..

[19] Kannan L., Shaw P.A., Morley M.P., Brandimarto J., Fang J.C., Sweitzer N.K., Cappola T.P., Cappola A.R. (2018). Thyroid Dysfunction in Heart Failure and Cardiovascular Outcomes. Circ. Heart Fail..

[20] Fiuzat M., Hamo C.E., Butler J., Abraham W.T., DeFilippis E.M., Fonarow G.C., Lindenfeld J., Mentz R.J., Psotka M.A., Solomon S.D. (2022). Optimal Background Pharmacological Therapy for Heart Failure Patients in Clinical Trials: JACC Review Topic of the Week. J. Am. Coll. Cardiol..

[21] Guo X., Yin H., Li L., Chen Y., Li J., Doan J., Steinmetz R., Liu Q. (2017). Cardioprotective Role of Tumor Necrosis Factor Receptor-Associated Factor 2 by Suppressing Apoptosis and Necroptosis. Circulation.

[22] Shanmugam G., Narasimhan M., Sakthivel R., Kumar R.R., Davidson C., Palaniappan S., Claybomb W.W., Hoidal J.R., Darley-Usmar V.M., Rajasekaran N.S. (2016). A biphasic effect of TNF-alpha in regulation of the Keap1/Nrf2 pathway in cardiomyocytes. Redox. Biol..

[23] Pingitore A., Landi P., Taddei M.C., Ripoli A., L’Abbate A., Iervasi G. (2005). Triiodothyronine levels for risk stratification of patients with chronic heart failure. Am. J. Med..

[24] Bashkin A., Saleh W.A., Shehadeh M., Even L., Ronen O. (2021). Subclinical hypothyroidism or isolated high TSH in hospitalized patients with chronic heart-failure and chronic renal-failure. Sci. Rep..

[25] Frey A., Kroiss M., Berliner D., Seifert M., Allolio B., Guder G., Ertl G., Angermann C.E., Stork S., Fassnacht M. (2013). Prognostic impact of subclinical thyroid dysfunction in heart failure. Int. J. Cardiol..

[26] Almandoz J.P., Gharib H. (2012). Hypothyroidism: Etiology, diagnosis, and management. Med. Clin. N. Am..

[27] Lee S., Farwell A.P. (2016). Euthyroid Sick Syndrome. Compr. Physiol..

[28] Iacoviello M., Guida P., Guastamacchia E., Triggiani V., Forleo C., Catanzaro R., Cicala M., Basile M., Sorrentino S., Favale S. (2008). Prognostic role of sub-clinical hypothyroidism in chronic heart failure outpatients. Curr. Pharm. Des..

[29] Kahana L., Keidar S., Sheinfeld M., Palant A. (1983). Endogenous cortisol and thyroid hormone levels in patients with acute myocardial infarction. Clin. Endocrinol..

[30] Lee S.Y., Rhee C.M., Leung A.M., Braverman L.E., Brent G.A., Pearce E.N. (2015). A review: Radiographic iodinated contrast media-induced thyroid dysfunction. J. Clin. Endocrinol. Metab..

[31] de Luca R., Davis P.J., Lin H.Y., Gionfra F., Percario Z.A., Affabris E., Pedersen J.Z., Marchese C., Trivedi P., Anastasiadou E. (2020). Thyroid Hormones Interaction with Immune Response, Inflammation and Non-thyroidal Illness Syndrome. Front. Cell Dev. Biol..

[32] Benites-Zapata V.A., Hernandez A.V., Nagarajan V., Cauthen C.A., Starling R.C., Tang W.H. (2015). Usefulness of neutrophil-to-lymphocyte ratio in risk stratification of patients with advanced heart failure. Am. J. Cardiol..

[33] Dunn G.P., Okada H. (2015). Principles of immunology and its nuances in the central nervous system. Neuro Oncol..

[34] Adelberg H.M., Siemsen J.K., Jung R.C., Nicoloff J.T. (1971). Scintigraphic detection of pulmonary bacterial infections with labeled thyroid hormones and pertechnetate. Radiology.

[35] Boelen A., Boorsma J., Kwakkel J., Wieland C.W., Renckens R., Visser T.J., Fliers E., Wiersinga W.M. (2008). Type 3 Deiodinase Is Highly Expressed in Infiltrating Neutrophilic Granulocytes in Response to Acute Bacterial Infection. Thyroid.

[36] van der Spek A.H., Jim K.K., Karaczyn A., van Beeren H.C., Ackermans M.T., Darras V.M., Vandenbroucke-Grauls C.M.J.E., Hernandez A., Brouwer M.C., Fliers E. (2018). The Thyroid Hormone Inactivating Type 3 Deiodinase Is Essential for Optimal Neutrophil Function: Observations from Three Species. Endocrinology.

[37] Pyzik A., Grywalska E., Matyjaszek-Matuszek B., Rolinski J. (2015). Immune Disorders in Hashimoto’s Thyroiditis: What Do We Know So Far?. J. Immunol. Res..

[38] van der Spek A.H., Fliers E., Boelen A. (2021). Thyroid Hormone and Deiodination in Innate Immune Cells. Endocrinology.

[39] Levy W.C., Mozaffarian D., Linker D.T., Sutradhar S.C., Anker S.D., Cropp A.B., Anand I., Maggioni A., Burton P., Sullivan M.D. (2006). The Seattle Heart Failure Model: Prediction of survival in heart failure. Circulation.

[40] Uthamalingam S., Patvardhan E.A., Subramanian S., Ahmed W., Martin W., Daley M., Capodilupo R. (2011). Utility of the neutrophil to lymphocyte ratio in predicting long-term outcomes in acute decompensated heart failure. Am. J. Cardiol..

[41] Acet H., Ertas F., Bilik M.Z., Akil M.A., Ozyurtlu F., Aydin M., Oylumlu M., Polat N., Yuksel M., Yildiz A. (2015). The relationship between neutrophil to lymphocyte ratio, platelet to lymphocyte ratio and thrombolysis in myocardial infarction risk score in patients with ST elevation acute myocardial infarction before primary coronary intervention. Postepy Kardiol Interwencyjnej.

[42] Sobolewska A., Wlodarczyk M., Stec-Michalska K., Fichna J., Wisniewska-Jarosinska M. (2016). Mean Platelet Volume in Crohn’s Disease Patients Predicts Sustained Response to a 52-Week Infliximab Therapy: A Pilot Study. Dig. Dis. Sci..

[43] Park J., Park J., Shin J.H., Oh Y.L., Jung H.A., Chung M.K., Choe J.H., Ahn Y.C., Kim S.W., Chung J.H. (2021). Prognostic Value of the Neutrophil-to-Lymphocyte Ratio before and after Radiotherapy for Anaplastic Thyroid Carcinoma. Cancers.

[44] Zhang G., Liao Y., Yang H., Tao J., Ma L., Zuo X. (2021). Irigenin reduces the expression of caspase-3 and matrix metalloproteinases, thus suppressing apoptosis and extracellular matrix degradation in TNF-alpha-stimulated nucleus pulposus cells. Chem. Biol. Interact..

[45] Stockwell B.R., Angeli J.P.F., Bayir H., Bush A.I., Conrad M., Dixon S.J., Fulda S., Gascon S., Hatzios S.K., Kagen V.E. (2017). Ferroptosis: A Regulated Cell Death Nexus Linking Metabolism, Redox Biology, and Disease. Cell.

[46] Chen Y., Fan H., Wang S., Tang G., Zhai C., Shen L. (2021). Ferroptosis: A Novel Therapeutic Target for Ischemia-Reperfusion Injury. Front. Cell Dev. Biol..

[47] Ye J., Zhang R.N., Wu F., Zhai L.J., Wang K.F., Xiao M., Xie T., Sui X. (2018). Non-apoptotic cell death in malignant tumor cells and natural compounds. Cancer Lett..

[48] Fearnhead H.O., Vandenabeele P., Berghe T.V. (2017). How do we fit ferroptosis in the family of regulated cell death?. Cell Death Differ..

[49] Shimada K., Skouta R., Kaplan A., Yang W.S., Hayano M., Dixon S.J., Brown L.M., Valenzuela C.A., Wolpaw A.J., Stockwell B.R. (2016). Global survey of cell death mechanisms reveals metabolic regulation of ferroptosis. Nat. Chem. Biol..

[50] Cao S.S., Kaufman R.J. (2014). Endoplasmic reticulum stress and oxidative stress in cell fate decision and human disease. Antioxid Redox Signal.

[51] Liang B., Liu L., Huang H., Li L., Zhou J. (2020). High T3 Induces beta-Cell Insulin Resistance via Endoplasmic Reticulum Stress. Mediat. Inflamm..

[52] Ashkenazi A., Fairbrother W.J., Leverson J.D., Souers A.J. (2017). From basic apoptosis discoveries to advanced selective BCL-2 family inhibitors. Nat. Rev. Drug Discov..

[53] Wu H., Zhu H., Zhuang Y., Zhang J., Ding X., Zhan L., Luo S., Zhang Q., Sun F., Zhang M. (2020). LncRNA ACART protects cardiomyocytes from apoptosis by activating PPAR-gamma/Bcl-2 pathway. J. Cell. Mol. Med..

