# Supplementary material for: Independent Association of Thyroid Dysfunction and Inflammation Predicts Adverse Events in Patients with Heart Failure via Promoting Cell Death
Source: J Cardiovasc Dev Dis. 2022 Aug 31;9(9):290. doi: 10.3390/jcdd9090290 (PMC9503390; doi:10.3390/jcdd9090290)
Supplement: Supplementary file 1 [file jcdd-09-00290-s001.zip › jcdd-1857894-supplementary.pdf]

Supplementary

Table S1 Interaction analysis of NLR, TT3 and FT3.

Variable	HR (95%CI)	p-value
**Model 1**		
TT3	0.51 (0.24-1.10)	0.085
NLR	1.17 (1.06-1.29)	0.002
TT3*NLR	0.89 (0.79-1.02)	0.084
**Model 2**		
FT3	0.74 (0.56-0.97)	0.030
NLR	1.13 (1.00-1.27)	0.044
FT3*NLR	0.98 (0.94-1.02)	0.397

Abbreviations: FT3 Free triiodothyronine; NLR, Neutrophil-to-lymphocyte ratio; TT3, Total triiodothyronine. Model 1 and model 2 were adjusted for age, sex, BMI, Amiodarone and LVEF.
